# OsTZF1, a CCCH-tandem zinc finger protein gene, driven under own promoter produces no pleiotropic effects and confers salt and drought tolerance in rice

**DOI:** 10.1080/15592324.2022.2142725

**Published:** 2022-11-18

**Authors:** Muhammad Ilyas, Safdar Hussain Shah, Yasunari Fujita, Kyonoshin Maruyama, Kazuo Nakashima, Kazuko Yamaguchi-Shinozaki, Asad Jan

**Affiliations:** a Institute of Biotechnology and Genetic Engineering, the University of Agriculture Peshawar, Khyber Pakhtunkhwa, Pakistan; b Japan International Research Center for Agricultural Sciences (JIRCAS), Tsukuba, Ibaraki, Japan; c Graduate School of Agricultural and Life Sciences, The University of Tokyo, Tokyo, Japan; d Research Institute for Agricultural and Life Sciences, Tokyo University of Agriculture, Tokyo, Japan

**Keywords:** CCCH-zinc finger protein, rice, senescence, stress, oxidative stress, RNA metabolism

## Abstract

Different abiotic stresses induce *OsTZF1*, a tandem CCCH-type zinc finger domain gene, in rice. Here, we report that transgenic rice plants overexpressing *OsTZF1* under own promoter (*P_OsTZF1_:OsTZF1-*OX [for overexpression]) transferred to soil showed normal growth similar to vector control plants. The *P_OsTZF1_:OsTZF1-*OX produced normal leaves without any lesion mimic phenotype and exhibited normal seed setting. The *P_OsTZF1_:OsTZF1-*OX plants showed significantly increased tolerance to salt and drought stresses and enhanced post stress recovery. Microarray analysis revealed a total of 846 genes up-regulated and 360 genes down-regulated in *P_OsTZF1_:OsTZF1-*OX salt-treated plants. Microarray analysis of *P_OsTZF1:_OsTZF1-*OX plants showed the regulation of many abiotic stress tolerance genes. These results suggest that *OsTZF1*-OX under own promoter show abiotic stress tolerance and produces no pleiotropic effect on phenotype of transgenic rice plant.

## Introduction

Zinc finger proteins are the most abundant and diverse families of plant regulatory proteins. Zinc finger proteins play an important role in certain cellular functions, containing transcriptional regulation, RNA binding, programmed cell death and protein–protein interactions.^1^ Various kinds of zinc finger proteins are classified on the basis of cysteine (Cys) and histidine (His) residues that binds to zinc ion. Different types of these proteins are C2H2, C2C2, C2HC, C2C2C2C2, C2HCC2C2, and CCCH.^2–4^

Tandem zinc finger proteins (TZF) are characterized by two similar C-x8-C-x5-C-x3-H motifs parted by 18 amino acids.^5^ Three Cys residues and one His is linked to the zinc ion and each CCCH zinc finger has the ability to bind to the 5`-UAUU-3` half site of the class II AU-rich element (ARE) 5`-UAUUUAUU-3`.^6–8^ In humans, *ZFP36, ZFP36L1*, and *ZFP36L2* belong to TZF gene family. Insulin, serum and other growth factors cause the expression of *ZFP36* also known as tristetraprolin (TTP).^7^ TTP binds to several important regulators such as tumor necrosis factor at the 3`-UTR of AREs and cause mRNA degradation.^9^ Tandem zinc finger proteins are also involved in epigenetic mRNA silencing by activating mRNA decay enzymes^10^ as well as nucleate processing bodies (PBs).^11,12^

A genome-wide survey of CCCH-type zinc finger genes in maize (*Zea mays* L.) identified 68 CCCH genes [*ZmC3H1-68*; ^13^]. Similarly, 34 and 91 CCCH family genes have been identified in *Medicago truncatula* and popular plants, respectively.^14,15^ Through computational analysis 68 and 67 CCCH family genes were identified in Arabidopsis and rice, respectively.^16^ These CCCH family genes were categorized into 11 sub-families in Arabidopsis and 8 subfamilies in rice.^16^ Arginine-rich (RR) tandem CCCH zinc finger (RR-TZF) group comprise of a large subfamily IX.^17^ Members in RR-TZF have been functionally characterized to function in seed maturation, germination and abiotic stress tolerance. *AtTZF1* regulates abscisic acid (ABA), gibberellin and sugar-mediated growth and stress responses.^18^ On the other hand, *AtTZF2* and *AtTZF3* are involved in jasmonate, ABA and oxidative stress responses.^19–21^ Other TZF genes such as *AtTZF4* (SOMNUS), *AtTZF6* (PEI1) and *AtTZF11/ AtTZF10* (*AtSZF1/AtSZF2*) function in light-dependent seed germination, embryogenesis and salt stress response, respectively.^22–24^ *OsDOS* (also known as *OsTZF2*) was reported to delay leaf senescence in rice.^25^ *OsTZF5* confers drought tolerance and increased grain yield under drought stress.^26^ *OsTZF1* was reported to be induced by drought and salt stress conditions and resulted in delayed leaf senescence.^27^

In this study the phenotype of *OsTZF1* overexpression plants driven by own promoter (*P_OsTZF1_:OsTZF1-*OX) were analyzed to examine if the *OsTZF1* own promoter could overcome the pleiotropic effects previously reported in *Ubi:OsTZF1-*OX rice.^27^ The *P_OsTZF1_:OsTZF1-*OX rice plants were also subjected to salt and drought stress to test if *OsTZF1* under own promoter confers tolerance against abiotic stresses.

## Material and methods

### Plant material and growth conditions

Rice (*Oryza sativa ssp. japonica* ‘Nipponbare’) was used in the present study. Initially, Seeds incubation was carried out at 42°C for 3 d in oven. Seeds were washed with sterilized distilled water and then sterilized with 0.2% HgCl_2_ solution. Seeds were rinsed five times with sterilized distilled water and then incubated on Murashige and Skoog (MS) media containing 25 mg/ml hygromycin. The germinated seedlings were transferred to soil for establishment and acclimatization under 12 hours light and dark cycles, flooded water and at 28°C.

### Senescence analysis of *P_OsTZF1_:OsTZF1* transgenic rice

For leaf senescence testing, leaf fragments (6 cm) from 6 weeks old *OsTZF1* transgenic (*Ubi:OsTZF1*-OX, *P_OsTZF1_:OsTZF1-*OX) and vector control plants were taken. The leaf fragments were incubated at 27°C for 4 d in petri plates containing water, different hormones or NaCl solution. Hormones included 10 µM methyl jasmonate (MeJA), 100 µM salicylic acid (SA) and 10 µM ABA solutions. The salt solution used was 250 mM NaCl. After 4 d, the difference in leaf coloring of different transgenic plants was observed.^27^

### Stress tolerance of *P_OsTZF1_:OsTZF1* transgenic rice

For high-salt stress, 4 weeks old *P_OsTZF1_:OsTZF1* transgenic plants and vector control grown in soil were subjected to 250 mM sodium chloride (NaCl) solution for 5 d. After salt stress treatment, plants were watered with fresh water for 2 weeks to check the survival rate. The surviving and continuously growing plants were counted and examined.^27^ For drought stress, 4 weeks old *P_OsTZF1_:OsTZF1* transgenic plants and vector control grown in soil were subjected to dehydration stress until the appearance of symptoms. After treatment, plants were watered for 2 weeks. The Plants maintaining continuous and suitable growth were counted and examined.^27^

### Transcriptomic analysis of *P_OsTZF1_:OsTZF1* transgenic rice

Microarray analysis was performed on 14 d old seedlings of the transgenic rice plants exposed to 2 days (2d) salt stress. Total RNAs were extracted from the harvested seedlings by TRIzole method/protocol. Cy3 and Cy5-labeled cDNA probes were made from the isolated total RNA. The probes were hybridized using the 44 K rice oligo microarray (Agilent Technologies). Briefly, two independent *P_OsTZF1_:OsTZF1* transgenic lines (OX#1 and OX#3) and one vector control were used. In each experiment, the reproducibility of the microarray analysis was measured by dye swapping. Using Feature Extraction software (version 10.10.1.1, Agilent Technologies), microarray slides were scanned and the data was analyzed after hybridization. Data analyses were carried out according to the Agilent methodology. Raw data was analyzed by Gene Spring GX software (version 11.5.1, Agilent Technologies). Lowess normalization method was used to normalize raw data.

### Real-time quantitative PCR (qRT-PCR)

Real-time quantitative PCR was performed for four (two up-regulated and two down-regulated) randomly selected genes identified in microarray experiment. RNA was isolated from transgenic rice leaves using TRIzole method. Synthesis of cDNA was performed from RNA through Superscript II reverse transcriptase using oligo dT primer. The qRT-PCR was done for three independent biological replicates of salt-treated *P_OsTZF1_:OsTZF1* transgenic rice and vector control with specific primers, using ABI 3700/ABI Studio quant and SYBR Green fluorescent dye chemistry (Takara, Japan). The primers used for up-regulated (Os01g0871600 & Os03g0587200) and down-regulated genes (Os07g014755 & Os10g0409400) are given in the following table.

**Table:** 

Gene IDs	Genes name	Primer type	Primer sequence
Os01g0871600	Peptide transporter (PTR2-B)	Forward primer	5’-CTCAGCAGCCTCCTCATCTC-3’
		Reverse primer	5’-AATCACAATTGCCATCAGCA-3’
Os03g0587200	Kinesin-like protein (KIN-12C)	Forward primer	5’-ACGAGATGGAGATGGAGACG-3’
		Reverse primer	5’-ATGCAGATGTAAACGCAGCA-3’
Os07g0147550	*OsPsbR2*	Forward primer	5’-ATGCAGGAAAGACAGGGTTG-3’
		Reverse primer	5’-TGTAAACCTTGCATGGCACT-3’
Os10g0409400	*OsBURP16*	Forward primer	5’-GAAGCCAGGTCAGAACAAGG-3’
		Reverse primer	5’-CGCACTGGAGAAGTGCAGTA-3’

### Statistical analysis

Statistical analysis was performed on the data obtained from salt and drought stress experiments. Statistical t-test was applied keeping level of significance less than 0.05 (P < .05).

## Results

### Phenotype of *P_OsTZF1_:OsTZF1* transgenic rice plants

**Figure 1.**
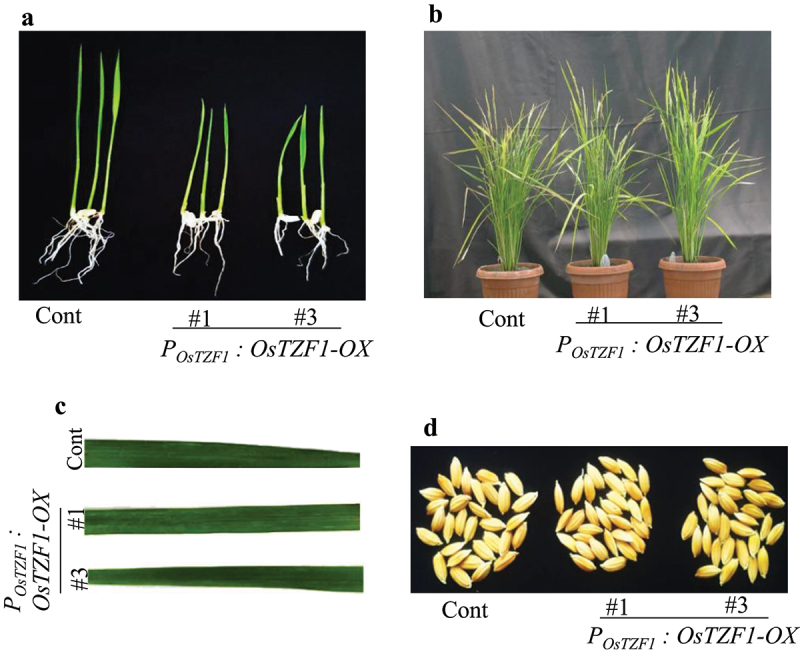
Phenotype of *POsTZF1:OsTZF1-*OX and control plants. A) Rice seedling growth of control and *POsTZF1:OsTZF1-*OX after 10 d of germination. B) Transgenic rice *POsTZF1:OsTZF1-*OX (#1 and #3), and control plants grown in soil exhibited similar phenotype. C) Leaves from control and P*OsTZF1:OsTZF1-*OX (#1 and #3) plants at seed setting stage. No brown lesions were observed. D) Phenotype of seeds harvested from control and *POsTZF1:OsTZF1-*OX (#1 and #3) at the time of harvest.

Ten days after seed germination, the growth of *P_OsTZF1_:OsTZF1*-OX seedlings was relatively slow compared to vector control (Figure 1a). The *P_OsTZF1_:OsTZF1*-OX seedlings transferred to soil showed normal growth and were similar in growth to vector control plants (Figure 1b). The *P_OsTZF1_:OsTZF1*-OX showed no difference in stature, number of tillers and number of panicle in comparison to vector control (Figure 1b). However, 8 weeks after heading (WAH) leaf yellowing phenotype appeared in vector control while *P_OsTZF1_:OsTZF1*-OX plants remained relatively green (data not shown). The leaf phenotype in vector control and *P_OsTZF1_:OsTZF1-OX* was similar and no brown lesions/spots were observed in case of *P_OsTZF1_:OsTZF1-*OX (Figure 1c). Furthermore, no difference in seed color of *P_OsTZF1_:OsTZF1*-OX and vector control plants was observed at the time of harvest (Figure 1d). It is concluded that *OsTZF1* gene driven under own promoter has no pleiotropic effect on the phenotype of rice.

### Delayed senescence exhibited by *P_OsTZF1_:OsTZF1* transgenic rice

**Figure 2.**
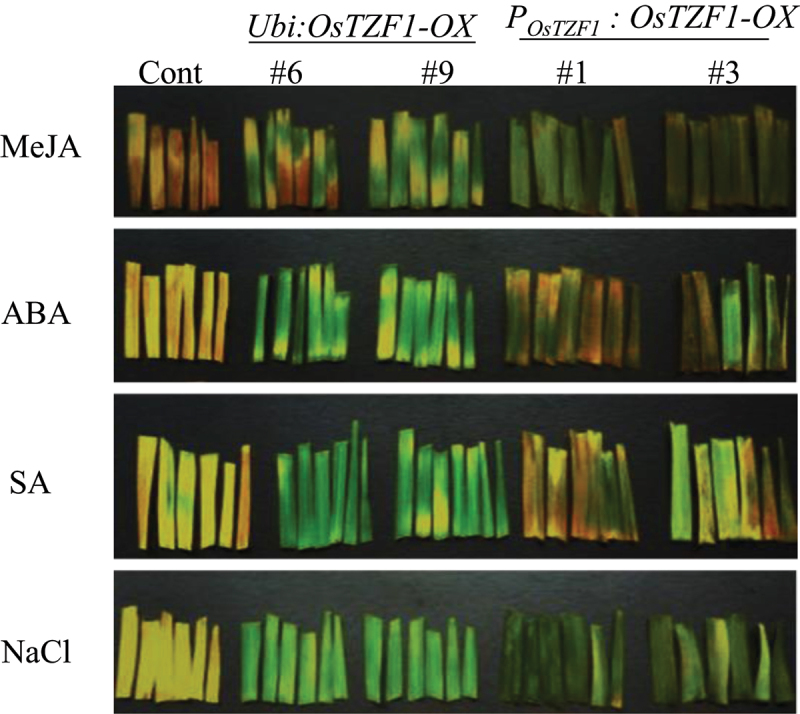
Delayed leaf senescence phenotype of *Ubi:OsTZF1*-OX and *POsTZF1:OsTZF1-*OX compared to controls under different senescence inducing conditions. Leaf fragments from the *OsTZF1*-OX and control plants were incubated in 10 µM MeJA, 10 µM ABA, 100 µM SA and 250 mM NaCl solutions in the dark for 4 d.

To study the role of *OsTZF1* gene in leaf senescence, leaf fragments (6 cm) from 6-week old *OsTZF1* transgenic (*Ubi:OsTZF1*-OX, *P_OsTZF1_:OsTZF1*-OX) and vector control were taken and examined for senescence. For 10 µM MeJA treatment, the leaf fragments of *Ubi:OsTZF1*-OX (#6 and #9) and *P_OsTZF1_:OsTZF1-*OX (#1 and #3) were compared to vector control. Under the treatment of 100 µM salicylic acid for 4 d in dark, the vector control leaf fragment appeared yellow while the leaf fragments of *Ubi:OsTZF1*-OX and *P_OsTZF1_:OsTZF1-*OX remained green (Figure 2). Similar results of delayed leaf senescence in case of *OsTZF1*-OX were observed under 10 µM ABA and 250 mM NaCl treatment (Figure 2). These results showed that *OsTZF1* driven under own promoter also delayed leaf senescence in rice compared to vector control.

### Salt and drought stress tolerance of *P_OsTZF1_:OsTZF1* transgenic rice

**Figure 3.**
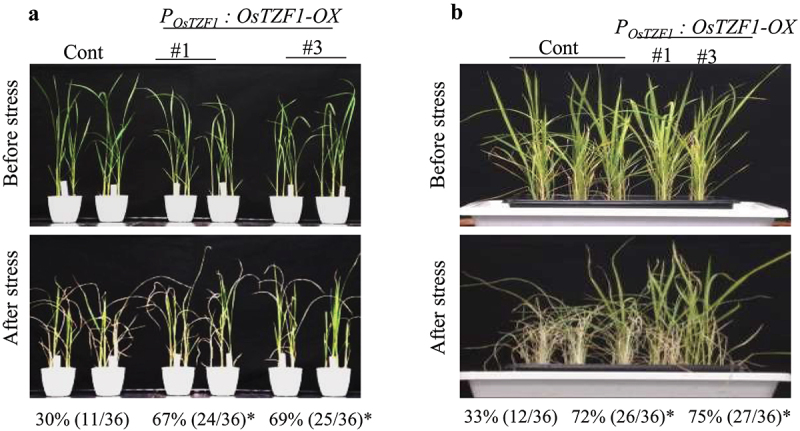
A) Salt-stress tolerance of *POsTZF1:OsTZF1-*OX *OsTZF1-RNAi* and control plants. The results are the average of three independent experiments with 12 plants per experiment. Asterisks indicate statistical significance (*, P < .050). B) Dehydration stress tolerance of *POsTZF1:OsTZF1-*OX, *OsTZF1-RNAi* and control plants. The results are the average of three independent experiments with 12 plants per experiment. Asterisks indicate statistical significance (*, P < .050).

Four week old plants grown in soil pots were irrigated with 250 mM NaCl solution for 5 d. The survival rates of *P_OsTZF1_:OsTZF1*-OX were significantly higher than vector control. About 67% *P_OsTZF1_:OsTZF1*-OX#1 and 69% *P_OsTZF1_:OsTZF1*-OX#3 plants survived, whereas the survival rate of vector control plants was 30% (Figure 3a).

Four week old plants grown in pots were exposed to drought stress. The plants were revived from drought stress by re-watering. Symptoms like leaf rolling and wilting appeared earlier in vector control compared to *P_OsTZF1_:OsTZF1-*OX. The survival rates of *P_OsTZF1_:OsTZF1*-OX plants were significantly higher than vector control. The survival rate for *P_OsTZF1_:OsTZF1*-OX#1 was 72% and *P_OsTZF1_:OsTZF1*-OX#3 was 75% compared to 33% in vector control (Figure 3b).

### Expression of stress related genes in *P_OsTZF1_:OsTZF1* transgenic rice

**Figure 4.**
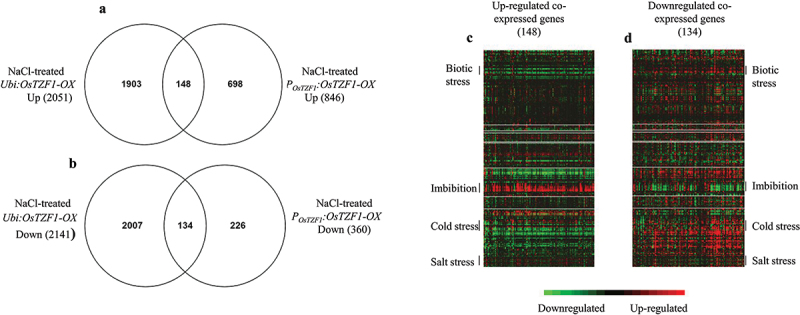
Comparative transcriptome analysis of *Ubi:OsTZF1-*OX and *POsTZF1:OsTZF1-*OX up-regulated and downregulated genes under high salt stress. A) Venn diagram of *Ubi:OsTZF1-*OX and *POsTZF1:OsTZF1-OX* up- regulated genes, where 148 genes were co-expressed. B) Venn diagram of *Ubi:OsTZF1-*OX and *POsTZF1:OsTZF1-* OX down-regulated genes, where 134 genes were co-expressed. C) Heat map of 148 upregulated genes co- expressed in *Ubi:OsTZF1-*OX and *POsTZF1:OsTZF1-*OX under 250 mM NaCl stress. D) Heat map of 134 downregulated genes co-expressed in *Ubi:OsTZF1-*OX and *POsTZF1:OsTZF1*-OX under 250 mM NaCl stress.

The *P_OsTZF1_:OsTZF1-*OX plants showed increased tolerance to salt stress as shown in Figure 3a. Transcriptomic analysis was carried out using two *P_OsTZF1_:OsTZF1-*OX lines (OX#1 and OX#3) and one vector control. Microarray analysis revealed that 1206 genes were regulated in *P_OsTZF1_:OsTZF1-*OX transgenic lines compared to vector control by two-fold or greater than two-fold under 2 d salt stress. Among 1206 genes, 846 were up-regulated and 360 were down-regulated in *P_OsTZF1_:OsTZF1-*OX salt stress treated plants. Previously, the microarray analysis of *Ubi:OsTZF1-*OX revealed the regulation of 4192 genes where 2051 genes were up-regulated and 2141 were down-regulated under 2 d salt stress.^27^ Comparative transcriptome analysis of up-regulated genes in *P_OsTZF1_:OsTZF1-*OX and *Ubi:OsTZF1-*OX revealed that 148 genes were co-expressed while 1903 genes and 598 genes were uniquely up-regulated in *Ubi:OsTZF1-*OX and *P_OsTZF1_:OsTZF1-*OX, respectively (Figure 4a). Analysis of down-regulated genes in *P_OsTZF1_:OsTZF1-*OX and *Ubi:OsTZF1-*OX showed that 134 genes were co-expressed while 2,007 and 226 genes were specifically down-regulated in *Ubi:OsTZF1-*OX and *P_OsTZF1_:OsTZF1-*OX, respectively (Figure 3b).

**Figure 5.**
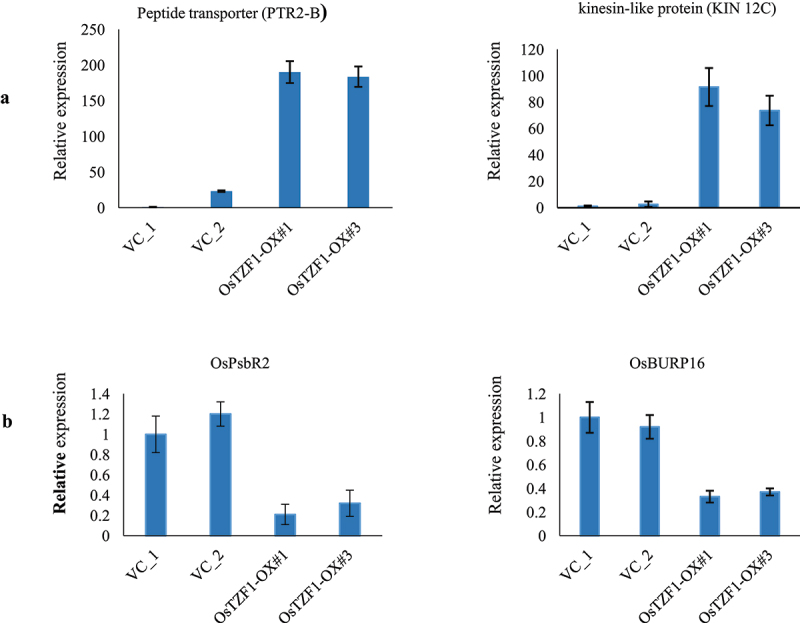
qRT-PCR analysis of up-regulated genes identified in *POsTZF1:OsTZF1-*OX plants by microarray analysis. A) The analyzed upregulated genes were peptide transporter (Os01g0871600) and kinesin-like protein (Os03g0587200). B) The downregulated genes analyzed were *OsPsbR2* (Os07g0147550) and *OsBURP16* (Os10g0409400).

The heat map of co-expressed up-regulated genes (148) in *Ubi:OsTZF1-*OX and *P_OsTZF1_:OsTZF1-*OX revealed that genes for biotic stress and cold stress were down-regulated while genes for imbibition and salt stress were up-regulated (Figure 3c). Similarly, heat map for co-expressed down-regulated genes (134) in *Ubi:OsTZF1-*OX and *P_OsTZF1_:OsTZF1-*OX revealed that genes for biotic stress and cold stress were up-regulated while genes for imbibition and salt stress were down-regulated (Figure 4d). To validate the microarray, two genes each from top 30 up or down-regulated genes were selected randomly. Results showed that peptide transporter (PTR2-B) and kinesin-like protein (KIN-12C) genes were up-regulated and *OsPsbR2* and *OsBURP16* were down-regulated in *P_OsTZF1_:OsTZF1-*OX comparison to vector control (Figure 5b).

## Discussion

Zinc finger proteins are involved in plant growth, development and stress responses through transcriptional regulation, RNA binding and protein–protein interactions.^1,28^ There are 67 CCCH zinc finger genes in rice divided into 8 subfamilies.^16^ Recently, functional studies on some CCCH zinc finger genes have been performed, but much remains to be revealed. Here, we report the effect of *OsTZF1* driven by own promoter in rice.

Previously, ^27^,reported that *Ubi:OsTZF1-*OX transgenic plants exhibited pleiotropic effects. *Ubi:OsTZF1-*OX plants showed reduced seed setting, delayed seed germination, retarded seedling growth, delayed leaf senescence, brown lesions on leaves and brownish seeds.^27^ In similarity to *Ubi:OsTZF1-*OX plants, the seedling growth in *P_OsTZF1_:OsTZF1-*OX plants was slow compared to vector control (Figure 1a). However, at subsequent stages such as mature vegetative or reproductive stages, the *P_OsTZF1_:OsTZF1-*OX transgenic and vector control plants showed similar phenotype (Figure 1b). Neither any difference was observed in seeds or leaves phenotype between *P_OsTZF1_:OsTZF1-*OX and vector control nor any brown lesions were observed on the leaves of *P_OsTZF1_:OsTZF1-*OX plants (Figure 1b–d). The ectopic overexpression of *OsDOS* showed several pleiotropic developmental phenotypes such as delayed growth, shorter stature, abnormally developed panicles, deferred heading and severe sterility, as well as delayed leaf senescence.^25^ Extremely stunted growth and reduced seed setting was observed in *Ubi:OsTZF5-*OX transgenic rice plants. However, expression of *OsTZF5* under stress-inducible *OsNAC6* promoter resulted in drought tolerance without negatively affecting growth in rice plants.^26^ Maize ubiquitin promoter is considered a constitutive promoter and has broad spectrum expression patterns. Putative *OsTZF1* promoter fragment is a stress inducible promoter. Previously, Jan et al., 2013, reported various *cis*-acting elements involved in the response to abiotic stresses in the 1,417-bp promoter region of *OsTZF1*.^27^ The identified *cis*-acting elements were five ABA responsive elements [ACGTG; ^29^], three MYB core sequences [CNGTTR; ^30^] and four recognition sites for MYC [CANNTG; ^31^]. The *OsTZF1* promoter also contained some putative *cis*-acting elements involved in the response to biotic stresses, including three WRKY71OS sequences [TGAC-containing W-box; ^32^] and seven W-boxes of different types, which are known as recognition sites for WRKY transcription factors. With the above information, putative promoter of *OsTZF1*was considered to be a good stress inducible promoter. Our results show that though *OsTZF1* expressed under own promoter has slow seedling growth in the initial 10 d after germination, it has no other negative effect on the phenotype of rice plants and confers abiotic stress tolerance (Figure 3a,b).

Under different phytohormones such as ABA, MeJA, SA or NaCl stress, delayed leaf senescence was observed in *P_OsTZF1_:OsTZF1-*OX and *Ubi:OsTZF1-*OX compared to vector control (Figure 2). Previously, it was reported that delayed leaf senescence was associated with tolerance to oxidative stress^33^. *Ubi:OsTZF1-*OX plants exhibited delayed leaf senescence under ABA, JA, SA, hydrogen peroxide and several abiotic stresses, showing that delayed senescence might be due to tolerance to oxidative stress.^27^ As described earlier, the putative promoter of *OsTZF1* is a stress inducible promoter, *P_OsTZF1_:OsTZF1-*OX also exhibited delayed senescence phenotype under hormones tested or NaCl. Further characterization of *P_OsTZF1_:OsTZF1-*OX transgenic plants may help to reveal its function under other stresses.

The *P_OsTZF1_:OsTZF1-*OX plants exhibited enhanced salt and drought tolerance compared to vector control plants. The survival rates of *OsTZF1-*OX plants were significantly higher than vector control plants after recovery from salt and drought stress (Figure 3). It was reported that overexpression of *OsDRZ1* increased drought tolerance in rice seedling. The transgenic plants had accumulated high free proline and less reactive oxygen species and had enhanced antioxidant enzymes activity.^34^ *OsTZF5* over expression could confer drought tolerance and increased grain yield under drought stress.^26^ Different transcription factors like OSISAP1, TFIIIA-type ZFP252, ZFP179, OsDREB1A, OsDREB1F and OsDREB2A have been reported that showed improved salinity and other abiotic stress tolerance in plants.^16,35–39^ Consistent with the previous results, our finding suggests that overexpression of *OsTZF1* under own promoter could enhance salt and drought stress tolerance in rice.

Transcriptomic analysis revealed that a total of 1206 genes with two-fold change were regulated in *P_OsTZF1_:OsTZF1-*OX, among which 846 genes were up-regulated and 360 genes were down-regulated under high salt stress. A total of 2,051 and 2,141 genes were up- and down-regulated, respectively, under salt stress in *Ubi:OsTZF1-*OX compared to vector control.^27^ Comparative transcriptome analysis of salt-treated *P_OsTZF1_:OsTZF1-*OX and *Ubi:OsTZF1-*OX revealed that 148 genes were co-expressed in up-regulated genes while 134 genes were co-expressed in down-regulated genes (Figure 4a,b). The heat map of co-expressed up-regulated genes revealed that genes for biotic stress and cold stress were down-regulated while genes belonging to biotic stress and cold stress were up-regulated in co-expressed down-regulated genes (Figure 4a,b). Jan et al., 2013, reported a contrasting expression pattern of genes to salt and drought stress response. The contrasting results indicate that *OsTZF1-*OX may show attenuated response to abiotic stresses (salt and drought stress).^27^ For example, the *atszf1-1 atszf2-1* double mutant also exhibited higher induction of stress-inducible genes compared to vector control and were less tolerant to high salt.^24^ Further detailed transcriptomic analysis may help to shed light on the attenuated stress response of *OsTZF1-*OX transgenic plants to abiotic stresses.

## Conclusion

*P_OsTZF1_:OsTZF1-*OX exhibited no pleiotropic effects previously observed in *Ubi:OsTZF1-*OX transgenic plants. Delayed leaf senescence was also observed in *P_OsTZF1_:OsTZF1-*OX compared to vector control under different stress inducing conditions. *P_OsTZF1_:OsTZF1-*OX plants exhibited high tolerance to salt and drought stress. The expression of *OsTZF1* under other stress-inducible promoters such as *Oshox24* is suggested to generate stress tolerant plants without any affect on plant growth and development.
